# Assessment of some factors that influence the productiveness and competitive performance of local pharmaceutical manufacturing companies in a low-income country setting: The case of Ethiopia

**DOI:** 10.1371/journal.pgph.0005631

**Published:** 2025-12-11

**Authors:** Yegnaneh Anley, Yeniewa Kerie Anagaw, Fasika Mekete Alemu, Ayenew Ashenef

**Affiliations:** 1 Department of Pharmaceutics and Social Pharmacy, School of Pharmacy, College of Health Sciences, Addis Ababa University, Addis Ababa, Ethiopia; 2 Department of Pharmaceutical Chemistry, School of Pharmacy, College of Medicine and Health Sciences, University of Gondar, Gondar, Ethiopia; 3 Department of Pharmaceutical Chemistry and Pharmacognosy, School of Pharmacy, College of Health Sciences, Addis Ababa University, Addis Ababa, Ethiopia,; 4 Advisor to the Minister, Ministry of Health, Addis Ababa, Ethiopia; Islamic Azad University South Tehran Branch, IRAN, ISLAMIC REPUBLIC OF

## Abstract

Local pharmaceutical production reduces dependency on imports. It also strengthens country-based medicine supply. In Ethiopia, there are government policies in place that aim to support local manufacturers. However, most of them operate below capacity, supplying only 15–20% of national pharmaceutical needs. This study assessed internal, external, and production-related factors that influence the productivity and competitive performance of local pharmaceutical manufacturing companies. A mixed concurrent triangulation design was conducted from October 2021 to February 2022. Quantitative data were collected from 40 employees across ten local manufacturers, while qualitative data were obtained through 29 key informant interviews with representatives from manufacturers, the Ethiopian Food and Drug Authority (EFDA), the Food, Beverage, Pharmaceuticals Industry Development Institute (FBPIDI), and the Ethiopian Pharmaceuticals and Medical Supplies Manufacturing Association (EPMSMA). Quantitative data were analyzed using SPSS version 25, and qualitative data were analyzed thematically. Internal factors, including material handling (β = 0.57, P < 0.036) and production planning and machine maintenance (β = 0.16, P < 0.047), were significantly associated with manufacturing performance. Among external factors, policy and economic conditions (β = -0.07, P < 0.042) significantly affected performance. Only three manufacturers fully complied with cGMP standards. Most government policies designed to support local production were not yet implemented, resulting in underutilization of capacity (<50%). The major challenges are limited foreign currency, technology transfer hurdles, shortage of qualified personnel, raw materials inadequacy and tax policies favoring imports. Both internal and external factors significantly influence the performance of local pharmaceutical manufacturers. Although government policy directions exist to support them, incomplete implementation limits their impact.

## Author summary

The study assesses the existing situations regarding local pharmaceutical manufacturing capacity and capability in Ethiopia. It identified material handling procedures, production planning, and machinery maintenance highly affecting the making of medicines in the country. It furthermore identified compliance with the standards, government policies as key factors that are affecting their performance. The paper highlighted the multifaceted challenges of local pharmaceutical manufacturing in low and middle-income countries as exemplified by Ethiopia. Hence, the solution to promote viable local manufacturing in Africa and other LMICs relies on the holistic approach that encompasses government, the companies, the health system, and international organizations working concurrently to solve the challenges highlighted in the study.

## 1. Background

The pharmaceutical industries are concerned with researching, designing, developing, and marketing efficacious, safe, and high-quality medicines to cure human and animal diseases. In doing so, they are not only improving the quality of life and alleviating suffering but also creating employment, helping in growing economies, and returning desirable profits to their shareholders [1]. Access to safe, effective, and affordable medicines remains poor in many developing countries [2]. Local pharmaceutical production of essential medicines is one of the ways that helps to ensure the supply of safe, efficacious, and affordable medicines [3].

Africa has about 500 drug manufacturers; however, they are continually challenged by underinvestment in the sector, infrastructure limitations, regulatory challenges, limited access to international markets, and donor-funded procurement requirements. Furthermore, only five of the 500 drug producers in Africa are certified by a global regulator or the WHO current international standards. This is unsustainable. Thus, the continent must act now to increase the sustainability and flourishment of medicine production systems [4].

Kenya has 38 pharmaceutical manufacturers [5], whereas Ethiopia currently has only eleven active local pharmaceutical manufacturing companies. None of those produces medicines against tuberculosis, HIV/AIDS, and malaria [6]. The essential drug demands are very high due to the increasing burden of communicable diseases (Malaria, HIV/AIDS, and TB), non-communicable diseases (Diabetes, Cancer, and Hypertension), rising healthcare coverage, and progressive growth of population [7]. Thus, the need for increased access to pharmaceutical products is a must [8]. However, according to World Bank statistics, manufacturing companies in Ethiopia, including those in the pharmaceutical sector, have experienced stagnation and declining profits over the past five years due to a challenging and unstable operating environment [9].

The government’s support of the sector is manifested by establishing and strengthening institutions like the Ethiopian Food and Drug Authority (EFDA), the Food, Beverage, and Pharmaceuticals Industry Development Institute (FBPIDI), and laying the groundwork for policies and incentives designed to encourage investment and development of the sector. Even though policy frameworks that support the industry’s growth by designing a strategic plan had been established by the government, until now, most of the local manufacturers operate below their capacity. They supply only about 15%-20% of the pharmaceutical market’s share with a limited portfolio range. The stated percent is covered from demands as calculated from human medicine consumption in the 2021/2022 year of the country. The remaining needs are covered by imported medicines from foreign pharmaceutical industries [10]. Moreover, more than 90% of the raw materials used in pharmaceutical production are imported, while only a few inputs are locally sourced. Most packaging materials, such as cartons and empty capsules, are also imported [9]. This adds more burdens, as Sub-Saharan African countries heavily rely on imports to meet their demand for essential medicines, allocating a significant portion of their financial resources to this area at the expense of other priorities such as food security, education, and energy supply [11].

The African Union and its Africa Center for Disease Control (AU CDC) had defined 8 bold programs for a sustainable vaccine manufacturing ecosystem: Market design &demand intelligence; access to finance; regulatory strengthening; technology transfer and intellectual proprietary protection (IP); Research & Development and Talent development; and Infrastructure setup and modernization. These bold programs would have formed a good basis for a comprehensive assessment of factors that influence pharmaceutical manufacturing companies’ efficiency in Ethiopia and elsewhere [12].

A study on the local pharmaceutical manufacturing sector in a low-income country indicates that capacity utilization and access to export markets have a significant impact on pharmaceutical performance, whereas regulatory policies and unplanned downtime have some effect [11]. Several related studies have identified competition, government regulations, international regulations, and production costs as key factors influencing the performance of pharmaceutical manufacturing. Additionally, a study on corporate brand equity and firm performance in the Kenyan pharmaceutical industry revealed that corporate reputation, firm competitiveness, value chain activities, and resource allocation significantly influenced organizational performance [13].

In Ethiopia, most of the local pharmaceutical manufacturing firms’ market share and their growth were found below the expectations set [10]. Most firms operate in environments that are potentially vulnerable to competitive actions, so they need effective production and marketing strategies. To the best of the investigators’ knowledge, there is a lack of published research work exploring the influence of production techniques and regulatory activities on pharmaceutical manufacturing companies in Ethiopia. In addition, internal and external factors that influence the performance of local pharmaceutical manufacturing companies have not been assessed till now. In response to the limited evidence and to fill this gap, this study aims to assess internal and external factors that influence the performance of local pharmaceutical manufacturing firms. This study also explores production techniques and regulatory oversights of local pharmaceutical manufacturing firms regarding CGMP standards.

## 2. Methods and materials

### 2.1. Study area

This study was conducted in currently active local pharmaceutical manufacturing factories within a 200 KM radius of Addis Ababa. Under this radius, we also included FBPIDI, EFDA, and the Ethiopian Pharmaceuticals and Medical Supplies Manufacturing Association (EPMSMA).

### 2.2. Study design and period

A mixed concurrent triangulation study design was employed from October 20, 2021, to February 10, 2022. It is based on performing both qualitative and quantitative data collection and analysis simultaneously. It helps to validate the research findings and leads to a comprehensive understanding of the research problem, as both methods were given equal weight.

A cross-sectional study design was employed for the quantitative, and a phenomenology design (understand and describe the lived experience of experts in the sector) approach was used for the qualitative part.

### 2.3. Source and study population

For the quantitative part, the source population consisted of top-level personnel (plant managers, department/unit heads, and senior sales/marketing staff) working in currently active local pharmaceutical manufacturing companies in Ethiopia. The study population included purposively selected key personnel from these firms who were directly involved in managerial, production, or regulatory functions relevant to the study objectives.

For the qualitative part, the source population included key personnel from local pharmaceutical manufacturing firms. In addition, representatives from the medicine registration and licensing directorate, medicine facility inspection directorate, and medicine quality assessment directorate of the EFDA were involved. Participants were also drawn from the industry pharmacy and quality administrative directorate, pharmaceutical and medical device industry development directorate, and pharmaceutical research and development directorate of the FBPIDI, as well as the chairman, secretary, and office head of the EPMSMA. Purposive sampling was used to select key informants from these institutions.

### 2.4. Sample size determination, sampling technique, and procedure

For the quantitative part, all currently active local pharmaceutical manufacturing firms located within a 200 km radius of Addis Ababa City were approached. Of the 11 eligible firms, 10 consented to participate; one company declined, and another situated beyond the 200 km radius and inactive during the study period was excluded. From each participating firm, four purposively selected key managerial personnel were included, yielding a total sample of 40 participants. This census-within-frame approach ensured adequate representation of managerial perspectives across firms. Moreover, for the qualitative study, key informants (KIs) were purposively selected from local pharmaceutical manufacturing firms, EFDA, FBPIDI, and EPMSMA. The final sample size was determined by data saturation, the point at which no new or relevant information emerged from additional interviews. Saturation was achieved after conducting 29 KI interviews.

### 2.5. Study variables

Company performance was the dependent variable of the study. While manufacturing downtime, production planning, and machine maintenance, material handling, technology upgrade, policy and economic factors, regulatory and legal provisions, challenges on technology, threat of new entrants, bargaining power of buyers and suppliers, threats of substitutes and competitive rivalry, cost leadership competitive strategy, differentiation competitive strategy, and focus competitive strategy were independent variables of the study.

The independent variables were measured using question items that had a Likert scale response range from 5 (strongly agree) to 1 (strongly disagree). In this study, the dependent variable company performance was measured by computation of four indicators, such as increase in company sales + market share + increase in innovation + company profitability, having 18 items with a dichotomous question**.**

### 2.6. Data collection tools and techniques

Self-administered questionnaires were developed and adapted from the literature for the quantitative part (S1 Text). Semi-structured and investigator-administered interview guides with probing questions were developed for an in-depth interview (S2 Text). An interview and audio records that took an average of 25–40 minutes were used for those who were willing, and a note was taken for those who refused to record their voice.

### 2.7. Data quality assurance

The data collection tools were pretested to check whether they could generate the desired information and to judge their internal consistency. One respondent from each local pharmaceutical industry participant was involved. The selection of each variable for the analysis was guided by previous literature and the objectives of the study. Normality of the dependent variable was assessed using the Shapiro-Wilk test and Q-Q plots. Linearity between independent and dependent variables was evaluated using scatter plots, and homoscedasticity was checked through residuals versus fitted values plots. The results indicated that all assumptions were reasonably met for the analysis. Additionally, Multicollinearity was checked using the variance inflation factor (VIF). Outliers and missing data were addressed, and the procedure for entering predictor variables was clearly described and applied. Moreover, the reliability of the items was checked by Cronbach’s alpha using SPSS software for the computation of internal consistency. As a rule, the researchers consider a measure to have adequate reliability if Cronbach’s alpha coefficient exceeds 0.70 [14].

For the qualitative part, three pilot interviews were conducted to check the relevance of the interview guide questions in terms of addressing study objectives. Based on these, minor amendments to the interview guide, such as the inclusion of additional probing questions and removal of vague and leading questions, were made.

### 2.8. Data analysis

The quantitative data were entered and analyzed using SPSS version 25.0. Variables having a P-value less than 0.05 were considered statistically significant. While qualitative data were analyzed manually using themes with verbatim quotes from the participants. The qualitative thematic analysis mainly followed a deductive approach. Qualitative data from open-ended questionnaire items were analyzed using thematic analysis. Transcripts were thoroughly checked for accuracy, coded inductively, and organized into themes and subthemes. The findings complemented the quantitative results, providing deeper insights into factors affecting the productivity and competitiveness of local pharmaceutical manufacturing companies.

### 2.9. Ethical approval and consent to participate

To conduct the study, ethical clearance was obtained on Ref.No: ERB/SOP/289/13/2021 from Addis Ababa University, School of Pharmacy Ethics Review Committee. An official letter from the School of Pharmacy, Department of Pharmaceutics and Social Pharmacy was written to the local pharmaceutical *manufacturing companies*, different governmental institutions, and for pharmaceutical industry associations to get consent for research conduct. For conducting the research, participants were, in prior, informed about the purpose of the study and what is expected from them. Participant’s involvement in the study was voluntary and withdrawal from the study at any time was possible. The participants were also assured that their answers would remain confidential and only findings were enclosed in the report without the use of personal identifiers.

Finally, oral informed consent has been taken from each participant and the information gathered from each selected category was kept confidential. Also, the obtained data were only used for the current study.

## 3. Results

### 3.1. Quantitative result

#### 3.1.1. Company profile.

The numbers of employees in the local pharmaceutical manufacturing companies varies from company to company. Accordingly, half 5 (50%) of the companies had about 100–200 employees, while 3 (30%) had 250–500 employees. One 1(10%) company had < 100 employees. Moreover, only one manufacturing company had > 500 employees which constitutes the largest size. Concerning the product type, the majority 7(70%) of the companies were involved in the production of human medicines/drugs, but 1(10%) was involved in both human and veterinary medicine, and 1(10%) was involved in veterinary vaccine &veterinary medicine. The remaining one (1(10%)) produces capsule shells. Regarding manufacturing lines, each 3 (30%) had one and two production lines. Two (20%) had four production lines, and 1(10%) have five production lines. Moreover, only 1(10%) had nine production lines which is the largest size.

With respect to the dosage forms they are manufacturing, large parenteral volume lines by2 (20%) of them. One (10%) had three dosage lines such as tablet, general capsule, and liquid lines, and the other 1(10%) had four dosage lines such as tablet, general capsule, liquid, and small volume parenteral lines. Additional one (10%) had four dosage lines such as a tablet, general capsule, liquid, and semisolid lines. The other one company (10 0%) had five dosage lines such as tablet, general capsule, liquid, small volume parenteral, and large volume parenteral lines. Moreover, 1(10 0%) had nine dosage lines such as a tablet, general capsule, liquid, beta-lactam (capsule, oral powder, and powder for parenteral) line, semisolid, and large and small volume parenteral lines. Besides 1(10%) had three dosage lines, including a tablet, a general capsule and also had veterinary bolus lines. Additionally, 1(10%) had a drug delivery capsule line, and the other one (10%) had three dosage manufacturing lines, including bacterial and viral veterinary vaccine, and veterinary bolus lines.

#### 3.1.2. Demography of respondents.

The socio-demographic profiles of respondents are described in Table 1. In this study, the majority, about 32(80%) of the respondents were male. More than two-thirds 27(67.5%) of the respondents were in the range of 31–40 years old. Regarding the professional experience of respondents, about 14(35%) employers had an experience of 3–6 years, and 3(7.5%) of them had an experience less than 3 years.

**Table 1:** Socio-demographic profile of respondents employed in a local pharmaceutical manufacturing company in Ethiopia, 2021.

Variable	Category	Frequency (%)	
		Quantitative study	Qualitative study
Sex	Male	32(80)	24(82.76)
	Female	8(20)	5(17.24)
Age in year	21-30	3(7.5)	2(6.89)
	31-40	27(67.5)	21(72.42)
	41-50	10(25)	4(13.80)
	>50	0(0.0)	2(6.89)
Work experience in year	<3	3(7.5)	3(10.3)
	3-6	14(35)	8(27.6)
	7-11	10(25)	9(31)
	>11	13(32.5)	9(31)
Educational Status	Diploma	1(2.5)	0(0.0)
	Degree	17(42.5)	13(44.83)
	Master and above	22(55)	16(55.17)
Field of study	Pharmacy	16(40)	11(37.9)
	Chemistry	1(2.5)	2(6.9)
	MSc in pharmaceutics	12(30)	8(27.6)
	MSc in pharmaceutical analysis	5(12.5)	5(17.2)
	MSc in pharmacology	1(2.5)	1(3.4)
	MSc in social Pharmacy	2(5)	1(3.4)
	Veterinary Medicine(DVM)	1(2.5)	1(3.4)
	MSc in business	2(5)	0(0.0)
Position	Plant manager	8(20)	5(17.2)
	Production manager	10(25)	6(20.7)
	Quality assurance manager	10(25)	9(31)
	R & D manager	2(5)	0(0.0)
	Sales/marketing senior staff	7(17.5)	0(0.0)
	Quality control manager	2(5)	0(0.0)
	Supply chain manager	1(2.5)	0(0.0)
	Senior expert	0(0.0)	4(13.8)
	Chairman association	0(0.0)	1(3.4)
	Secretary association	0(0.0)	1(3.4)
	Office head	0(0.0)	1(3.4)
	Director	0(0.0)	2(6.9)
Total		40(100)	29(100)

Regarding the educational status of respondents, more than half, 22(55%) of the respondents had master’s degrees. Additionally, 16(40%) of the respondents studied a first degree in pharmacy. Among all, 36(90%) of the respondents studied pharmacy disciplines. The highest numbers of respondents were delegated to production manager and quality assurance head positions. The lowest number of respondents was delegated to the supply chain head position (Table 1).

#### 3.1.3. Manufacturing performance indicators.

In this study increase in sales, profitability, increased innovation, and an increase in market share were major manufacturing performance indicators. Four indicators to measure the performance of manufacturing companies were computed, and their responses were summarized below.

i Increasing company sale

As described in Table 2, most of the respondents,34(85%), mentioned that increasing of product portfolio was the main factor that increases sales, followed by making work more efficient with technologies and increasing sales force knowledge by organizing training and meetings, as mentioned by 32(80%) of respondents. The remaining 31(77.5%) of them mentioned developing a customer sales structure that will also lead to an increase in sales.

**Table 2:** Key Factors Influencing Performance of Pharmaceutical Manufacturing Companies in Ethiopia, 2021 (N = 40).

Variables	Frequency (%)
**Factors Increasing Pharmaceutical Company Sales**	
Increasing the product portfolio of the company	34(85)
Making work more efficient with technologies	32(80)
Developing customer sales structure	31(77.5)
Increasing sales force knowledge through training	32(80)
**Factors Increasing Market Share**	
The firm brings to market a new technology ahead of its competitors	20(50)
Strengthening customer relations and protect existed market share	28(70)
Company obtained a GMP certificate	28(70)
Produce appropriate quality products, affordable prices, promotion, and distribution strategy	34(85)
**Factors Contributing to Increased Innovation of the company**	
Does your company invest in innovation and creativity to adapt a differentiation strategy?	29(72.5)
Using GMP as a production tool	25(62.5)
In-house research and development	19(47.5)
Working with universities and public research institutes	27(67.5)
Appropriate staff training to apply the technologies	17(42.5)
Investments by the company for innovation and creativity	23(57.5)
**Factors Increasing Company Profitability**	
Is your company profitable	33(82.5)
Appling GMP as a production technique tool	25(62.5)
Proper market competition for domestic pharmaceutical companies	23(57.5)
Following the situation of external and internal environment	25(62.5)
Revising production steps regularly to develop a product portfolio	23(57.5)

ii Market share of the company

Most of the respondents 34(85%), mentioned that producing appropriate quality products at affordable prices, promotion, and distribution strategy design were the most important factors to increase in market share of the company, as shown in Table 2.

iii Increased innovation

The majority 29(72.5%) of the respondents reported that their company investments in innovation and creativity to adapt a differentiation strategy contribute to the increment of innovations, followed by working with universities and public research institutes 27 (67.5%). Additionally, about 19(47.5%) of the respondents mentioned in-house research and development department or unit was the backbone for the increment of company innovations (Table 2).

iv Company profitability

Most of the respondents 33(82.5%) reported that their company was profitable. While 23(57.5%) of the respondents mentioned that proper market competition and revising production steps regularly to develop product portfolios were the main factors in increasing the profitability (Table 2).

#### 3.1.4. Descriptive analysis.

Several internal and external factors had an effect on the performance of pharmaceutical manufacturing companies. The extent to which these different factors influence the performance of manufacturing pharmaceutical companies was measured using a Likert scale of 1–5, where each number represents 5- strongly agree, 4-agree, 3- neutral, 2- disagree, and 1- strongly disagree. The results were analyzed based on the output values calculated. I fit falls in 1-1.80, it was determined as very low, 1.80-2.60 low, 2.60-3.40 medium, 3.40-4.20 high, and 4.20-5.00 very high.

Among the internal factors, material handling had a grand mean of 4.2 ± 0.64, followed by production planning and machine maintenance (3.93 ± 0.86). Among the external (macro-environment) factors, policy and economic factors, regulatory and legal provision, and technology challenges have a grand mean of 3.93, 3.83, and 3.52, with a standard deviation of 0.73, 0.81, and 0.92, respectively. Additionally, among the competitive strategy factors, cost leadership competitive strategy and differentiation competitive strategy had a grand mean of 3.65 and 3.7, with a standard deviation of 0.82 and 0.94, respectively.

The descriptive statistics of external and internal factors affecting pharmaceutical manufacturing performance are listed in Table 3. Hence, material handling, policy and economics, and timely production planning and machine maintenance factors were the top factors affecting the manufacturing performance, followed by manufacturing downtime and regulatory and legal provision factors. In contrast, the threat of substitute and competitive rivalry, bargaining power of buyer and supplier, and focus competitive strategy were the least factors affecting the manufacturing performance.

**Table 3:** Descriptive statistics of external and internal factors influencing manufacturing performance of local companies in Ethiopia, 2021 (N = 40).

Factors/ variables	Mean ±Std. Deviation	N	Rank
Manufacturing down time	3.87 ± 0.70	40	4
Production planning and machine maintenance	3.93 ± 0.86	40	3
Technology upgrade	3.48 ± 0.86	40	9
Material handling factors	4.2 ± 0.64	40	1
Policy and economic factors	3.93 ± 0.73	40	2
Regulatory and legal provision	3.83 ± 0.81	40	5
Challenges on technology	3.52 ± 0.92	40	8
Bargaining power of buyer and supplier	3.17 ± 0.81	40	12
Threat of new entrant	3.24 ± 0.88	40	10
Threat of substitute and competitive rivalry	3.08 ± 0.82	40	13
Cost leadership competitive strategy	3.65 ± 0.82	40	7
Differentiation competitive strategy	3.7 ± 0.94	40	6
Focus competitive strategy	3.22 ± 0.83	40	11

#### 3.1.5. Correlation analysis.

According to Cohen, an interpretation of the range of the coefficient of correlation has been grouped into the following: -0.3 to + 0.3 weak; -0.5 to -0.3 or 0.3 to 0.5 moderate; - 0.5 to - 0.9 or 0.5 to 0.9 strong; and -0.9 to-1 or 0.9 to 1 very strong [15]. In this study, a strong positive relationship was found between material handling (r = 0.65, p < 0.01), policy and economic factors(r = 0.59, p < 0.01), challenges on technology (r = 0.57, p < 0.01), and factors dealing with technology upgrade (r = 0.53, p < 0.01) with manufacturing performance. Similarly, there is a moderate positive relationship between competitive differentiation strategy (r = 0.48, p < 0.01), focus competitive strategy (r = 0.41, p < 0.05), bargaining power of buyer and supplier (r = 0.40, p < 0.01), threat of new entrant (r = 0.35, p < 0.05) and cost leadership competitive strategy (r = 0.32, p < 0.05) with manufacturing performance. Contrarily, there was no significant relationship between manufacturing downtime, regulatory and legal provision, production planning and machine maintenance, and the threat of substitute and competitive rivalry with company performance.

#### 3.1.6. Regression analysis.

Table 4 shows the multiple linear regression analysis. This was conducted to identify the effect of internal and external factors on the manufacturing performance of pharmaceutical companies. The regression result showed that there is a strong correlation between internal and external factors, with a performance at a value of 0.59. The R-squared value is 0.35, which implies that a 35% variation in manufacturing performance is explained by both internal and external factors. The remaining 65% of the variation in manufacturing performance is explained by other factors not included in this study. From the internal factors included in the model, material handling (β = 0.57, P < 0.036) and production planning and machine maintenance (β = 0.16, P < 0.047) had a significant effect on the manufacturing performance of local pharmaceutical firms. Similarly, among the external factors included in the regression model, policy and economic factors (β = -0.07, p < 0.042) had a significant effect on the manufacturing performance of local pharmaceutical firms. The above-mentioned factors significantly affect the manufacturing performance of pharmaceutical firms (Table 4).

**Table 4:** Regression analysis of factors influencing manufacturing performance of a local pharmaceutical company in Ethiopia, 2021 (N = 40).

Factors	Unstandardized β	Standardized β	Significance	95% CI	VIF
Constant	19.28		0.007	(5.74,32.83)	
Regulatory and legal provision	0.03	0.04	0.081	(0.36, 0.78)	1.3
Differential competitive strategy	0.34	0.38	0.371	(0.22, 0.91)	3.7
Cost-leadership competitive strategy	0.42	0.41	0.158	(0.16, 1.01)	3.1
Threats of substitutes and competitive rivalry	-0.01	-0.02	0.952	(-0.47, 0.45)	2.5
Material handling	0.54	0.57	0.036	(0.10, 0.72)	3.4
Bargaining power of buyer and supplier	-0.27	-0.26	0.325	(-0.81, 0.28)	2.7
Challenges on technology	-0.07	-0.09	0.741	(-0.49, 0.35)	2.9
Policy and economic factors	-0.04	-0.07	0.042	(-0.43, 0.33	3.4
Technology Upgrade	0.18	0.23	0.061	(0.08, 0.26)	2.9
Manufacturing downtime	-0.08	-0.12	0.082	(-0.42, 0.26)	2.6
Production planning and machine maintenance	0.15	0.16	0.047	(-0.39, 0.70)	2.9
Focus competitive strategy	0.26	0.26	0.314	(-0.26, 0.79)	2.6
Threat of new entrant	0.11	0.14	0.633	(-0.35, 0.57)	3.2
R	0.59				
R^2^	0.35				
Adjusted R^2^	0.18				
F- Statistic*	1.05				
Dependent variable	Performance				

### 3.2. Qualitative result

#### 3.2.1. Demographic profile of key informants.

A total of 29 respondents participated in the qualitative study. Among these 20 of them were from pharmaceutical manufacturing companies, 3 of them were from government supportive organization, i.e., FBPIDI, 3 of them were from regulatory, EFDA, and the rest 3 were from manufacturers association, EPMA. The socio-demographic information about participants’ is shown on the table (Table 1).

#### 3.2.2. Issues found in qualitative interviews.

The issues that had emerged in the qualitative analyses can be summarized in the following six perspectives as:

I) Issues related to Local companies’ production techniques compliance with the CGMP standards.
II) Challenges faced by the companies to comply with CGMP standards
III) Companies compliance status with the national regulatory, i.e., EFDA CGMP standards
IV) The role of Government strategic plan and policy in puts on local companies
V) Major operational challenges currently faced by local companies
VI) Government’s regulatory and supportive establishments self-audit assessments of the impact of policies set for the local companies.

Additionally, issue I: had six categories that concern with premises, utilities, quality control practices, sanitation and hygiene, training and documentation. In the perspective VI the assessed establishments from the regulatory, government support authority and the manufacturers association had expressed their perspectives about the current state of local pharmaceutical manufacturing.

Issue I: Production techniques compliance with CGMP standards:

Key informants from the companies expressed that they are handling pharmaceutical manufacturing per the CGMP guidelines with main components of GMP standards that deals with premises, utilities, quality control, sanitation and hygiene, training and documentation. Nevertheless, some key personnel had expressed difficulties in fulfilling CGMP standards as exemplified in the following quotes:

“*…... No skilled engineers in the local pharmaceutical sector which lead to spending more money to get foreign experts for timely calibration to check the performance of the AHS* (***Air Handling systems***) *is also an issue that needs priority intervention…..” KI 02*

*“The audit deficiency on the water purification system was investigated upon GMP audit assessment…..In line with this, the welding was not done by certified expertise like oriental welding thus there will be downtime because the company has no other space for the installation of a water system.”* KI 03

*“…emphasized that experienced staff turnover as a major problem to implement the level of QC practices”. KI 04 and KI 05*

Issue II: Challenges to comply with the CGMP standards:

The local pharmaceutical companies in Ethiopia are still not accredited to CGMP standards by international or stringent regulatory authorities. Only three of them are accredited to the CGMP standards by the national regulatory authority, EFDA at the time of this study. Key informants had expressed the challenges they face in complying with the CGMP standards as exemplified by the following quotes:

*“The absence of local experts for calibration of different instruments, such as thermo-hygrometers pressure gauge, pH meter, conductive meter, and HAVAC system was the major challenge for GMP implementation……”. KI 09*

*“Due to shortage of foreign currency purchasing raw materials, quality control reagents, reference, and equipment accessories are delayed. That is a headache to perform production-related activities validation…..”.. an opinion expressed by KI 10 and 11 Plant managers of two companies*

*Issue III: National medicine regulatory authority demand for CGMP compliance*

The EFDA had its requirements for establishing and operating pharmaceutical manufacturing in Ethiopia for human medicines and the Ethiopian Agricultural Authority (EAA) had also the same protocols for veterinary medicines. All local companies are urged to work fulfilling the CGMP standards set in the country based from criteria’s that are mainly adopted from the WHO/WOAH system. Nevertheless, currently the authorities did not shut up or close companies for not fulfilling such requirements except when default product(s) release on the market and subsequent recalls due to pharmacovigilance reports. However, it is in both authorities plan to implement that all companies should full fill their CGMP standards to continue operation in the country for all local companies. Thus, local companies are working hard to fulfill the regulatory authorities’ CGMP standards. There is CGMP master plan in place. Moreover, few companies are working to be accredited by international bodies too. This is described by the key informants as noted in the following quotes:

*“We have complied with the EFDA GMP certification and have taken the initial assessment inspection from WHO and we obtained some feedback for improvement. Now we are working to implement the WHO prequalification certificate” KI 11, 12, 13 Plant managers of three companies*

*“The inspectors from EFDA come regularly and inspected us. We export our products abroad; some of our precious customers come to our site and inspected us per international GMP principles before they make an agreement to purchase our products’‘ KI 13,14 Two respondents*

Issue IV: Strategic plan and policies related matters:

The Ethiopian government had come up with a strategic plan for local pharmaceutical production supported technically by WHO as “National Strategy and Plan of Action for Pharmaceutical Manufacturing Development” (2015-2025)” [8]. It had also included pharmaceutical manufacturing as key component of government economic growth plans at different national economic growth planning times. It had set measurable goals to achieve the sated up objectives. However due to many underlying challenges, none of the sated major goals were fulfilled till now. Key informants had expressed that even though the plans and policies are helpful in supporting their growth, still there are some bottlenecks to be addressed. Some exemplary descriptions of such bottlenecks were mentioned by the following statements from the key informants:

*“We are aware of the motivation package such as duty tax-free to import inputs and raw materials and facilitated as per the government directive. In addition, the FBPIDI provides supporting letters to get hard currency and to tax office for tax-exempt applications as per the second schedule tariffs procedure. Moreover, the governmental pharmaceutical supplied agency (EPSA) gives a 25% price preference privilege for procurement than the foreign pharmaceutical suppliers. It also provides 30% advance payment…. “KI 15*

*The constraint on duty and tax-free policy to import raw material is not mentioned in the second schedule tariffs procedure. For example, talcum, polyvinyl chloride (PVC), reagents, reference standards, and spare parts are taxable. The second constraint is foreign exchange…. When anyone goes to any bank, the meaning of medicine is on the finished goods, not the raw material. Medicine is considered a top priority by this priority policy incentive, so the importers have a better priority than us. Rather we are considered as manufacturers which is the third priority…,”. KI 16*

Issue V: Operational challenges

The key informants had stated that internet interruption, power fluctuation, staff turnover, and lack of experts were the main operational challenges in pharmaceutical production that the companies face frequently.

*“The main challenge is lack of experts’; expert in the pharmaceutical engineering area and staff turnover (chemist and pharmacist) in a highly competitive industry. Moreover, the power fluctuation and electricity, as well as internet interruption, were very high”. KI 18*

*“During export of our products, it needs special logistic transport packaging. Since the substance is extremely sensitive to uncontrolled temperature and humidity, it is transported in refrigerated containers. While the Ethiopian shipping lines do not have such type of facilities, because of these we are forced to freight transport our product by air freight….. as it is very well known the air freight charge is very expensive, thus it did not make us competitive with other giant company”. KI 19 The plant manager*

Issue VI: Government regulatory and supportive establishments’ assessment of the policies usefulness for the local companies

The FBPDI had been established to support local pharmaceutical manufacturers. The regulatory authority even though its main focus is regulation, it practiced supportive regulation and consultancy to establish a viable local pharmaceutical manufacturing companies in the country. The local manufactures had also established their own association to deal with the government in relation to policy or other issues they might face in their day to day operations. Both organizations had stated that still many challenges and issues had to be addressed by government in order to nourish and flourish local pharmaceutical manufacturing in Ethiopia by addressing the current challenges faced by them. They corroborated the challenges as mentioned in the following quotes:

*“From our side, the authority uses standard regulatory procedures to ensure quality products for market accessibility by considering cGMP status, dossier compilation, and lab results. Even though most of the local manufacturers didn’t fulfill all criteria, the authority considers lab results to sell the batch/lot. We also encourage a local company to fulfill the registration criteria. One of the basic challenges for the regulations is the distribution of substandard and counterfeit products in the market…it is not only in Ethiopia it is a global issue We give priority to local manufacturer registration; provide the necessary support, consultancy, and training on regulatory issues. In addition, different type of recall was investigated in the last 5 year including poor packaging, leakage of large volume parenteral, and fractured tablet”. KI 20*

*“The obstacle to utilizing policy advantage to increasing the market share is the National Bank FOREX Directive 67/2020 (don’t consider local Pharmaceutical manufacturing companies as priority sector), poor taxation system, lack of priority and the waiting of manufacturer in the queue at (Customs, shipping lines, Ethiopian Airlines, and other government offices). The majority of policies favor import rather than local Pharmaceutical manufacturing companies. That is why the number of importers is increasing dramatically. Some of local pharmaceutical manufacturing companies are on the verge of closing as happened between 2000 to 2007G.C when more than 4 pharmaceutical manufacturing companies closed.…”. KI 23*

## 4. Discussion

This study aimed to assess the influence of internal and external factors that affect the performance of local pharmaceutical manufacturing firms in Ethiopia. It also explores major constraints, the influence of production techniques, and regulatory and legal provisions on local pharmaceutical manufacturing firms. Local Pharmaceutical manufacturing’s critical importance was manifested during Africa’s response to the COVID-19 pandemic. The pandemic had exposed the inherent weaknesses in Africa’s health systems. Today, 80–90% of global HIV, TB, and Malaria cases occur on the continent. But, Africa yet imports ~80% of its medicine needs funded by international donors at an estimated $14 billion a year [16].

Africa is capable of manufacturing its own medicines. The partnership between Africa CDC and Medicines for Malaria Venture (MMV) will increase local medicine manufacturing. It focuses on establishing several regional hubs to manufacture Active Pharmaceutical Ingredients (API) and Finished Pharmaceutical Products (FPP). The collaboration will support African Union Member States and is aimed at accelerating and scaling up African manufacturing, building on existing capacities and developing new ones to support the manufacture of quality-assured malaria APIs and FPPs [17].

To complete the implementation of continental pharmaceutical manufacturing action plan (PMPA)and establish a robust pharma manufacturing sector in Africa, the continent must redouble its efforts in capacity-building, knowledge transfer, cross-sector coordination, and rigorous implementation of the African free trade act (AfCFTA). It should further mobilize financial resources from international financial institutions and development banks; and encourage cross-country collaboration to strengthen human capital development funding. Only then Africa can close the implementation gap and accelerate the development of Africa’s pharmaceutical industry [16,18].

In correlation analysis, a strong and positive relationship was found between material handling, policy and economic factors, challenges on technology, and technology upgrade factors with manufacturing performance. These findings are in line with a previous study that reported a moderate positive relationship between competitive differentiation strategy, focus strategy, bargaining power of buyers and suppliers, threat of new entrants, and cost leadership strategy with manufacturing performance [19]. This finding also coincides with the results of different studies that noted the government regulations significantly determine the growth of a firm [20]. However, some findings showed that government policy and regulations are weak explanatory variables for firms’ performance [21]. On the importance of government incentives, the study findings noted that government interventions through incentives and subsidies are particularly important for the development of local companies [22]. A study conducted in Kenya revealed that the majority of respondents (67%) confirmed that the government provides incentives to local pharmaceutical manufacturing firms [13].

In the multiple linear regression analysis, material handling and production planning, and machine maintenance were internal factors that had significant effects on the manufacturing performance of local pharmaceutical firms. Material handling was significantly associated with the manufacturing performance of local firms [23]. This finding was also in line with a WHO guideline that proper material handling focused on increased output and the subsequent cost reduction through the use of personal protective equipment in the handling of unsafe raw materials [24]. Additionally, a study conducted on the influence of material handling practices on the performance of large-scale manufacturing firms in Nairobi, Kenya, found that material handling automation had an impact on the large manufacturing firm’s performance [25,26].

In this study, production planning and machine maintenance also showed a significant and positive association with the performance of local pharmaceutical firms. The use of a well-applied system of maintenance and design of maintenance-oriented systems allows for decreased downtime, production losses, and reduced costs [27]. However, according to a study conducted in Kenya, raw materials, labor, machinery, fuel/electricity, and taxes were identified as the main drivers of drug production costs. Specifically, 90% of respondents cited imported raw materials and machinery as major cost factors, 83.3% mentioned labor, all respondents identified fuel/electricity, and 96.7% indicated taxes as significant contributors to high production costs [13]. The possible reason for the association could be a good maintenance plan, one that is integrated with the production plan and can result in considerable savings. This integration with production is crucial because production and maintenance have a direct relationship. Any breakdown in machine operation results in disruption of production and leads to additional costs due to downtime, loss of production, decrease in productivity and quality, and inefficient use of personnel, equipment, and facilities [28].

This finding is supported by a previous study that states the success of manufacturing companies heavily depends on production performances, in terms of increased availability by optimizing and improving maintenance processes [29]. In addition, production planning satisfies the requirements of the marketing department through the provision of quantities and products on demand at the highest level of manufacturing efficiency. After formulation, the production planning should be strictly and constantly followed for the effectiveness of the actual manufacturing performance [30]. It is supported by a study done in Zimbabawe that machine maintenance is paramount important since it provides a means of plant and equipment maintenance, thereby enhancing the manufacturing process [31]. This finding is also supported by studies done on the assessment of the effectiveness of maintenance-oriented design [32], a study done in Kenya [33], and a study done in Netherlands [28].

According to the qualitative findings, the local pharmaceutical manufacturers point out that preventive maintenance is done once a year, and breakdown maintenance is done when needed in the engineering department to avoid unplanned downtime to enhance performance. Among the external factors included in the regression model, policy and economic factors have a significant effect on the manufacturing performance of local pharmaceutical firms. Most of the efforts to improve the regulation of medicines in Africa are hampered by the lack of clear policies or legal and regulatory frameworks for regulating medicines at the national as well as regional levels. The African Union model law on medical products regulation and harmonization was adopted in 2016 and aims at addressing legislative gaps that hamper effective regulation of medicines to promote local production of pharmaceuticals, with a view to the protection of public health and contribution to economic growth [34].

Our findings verified that the government has been willing to make sure the proper regulations are in place to support local manufacturing, disregarding the challenges associated with implementation. This was in line with a study conducted in Zambia [35]. From the qualitative findings, the participants from EPMSMA mentioned that the government has developed a clear and supportive policy for local manufacturers since 2016. But a majority of policies were not enacted or implemented. Hence, there are lots of hurdles at various places. The association also pointed out that the majority of policies were not implemented, and the local pharmaceutical manufacturing companies are on the verge of collapse and are utilizing below 50% of their capacity. Hence, the policy favors import over local pharmaceutical manufacturing companies despite the increase in pharmaceutical demand. The lack of foreign currency to import raw materials from abroad and the inflation rate reduced the production capacities of local pharmaceutical manufacturer firms [35]. This investigation is supported by a previous study that indicated that in developing countries, pharmaceutical manufacturers import most of the raw materials, equipment, and spare parts from abroad, thereby utilizing foreign currency, which will affect the production performance [36]. Additionally, a similar study in Ethiopia revealed that commonly reported challenges include limited access to foreign currency, inconsistent supply of input materials, inadequate physical infrastructure, and a shortage of qualified personnel [11].

According to this study, the local pharmaceutical supply is very far from the expectations of the government’s growth strategic plan I &II goals; the growth of the pharmaceutical industry is retarded. This aligns with a study indicating that the South African pharmaceutical industry is small, financially fragile, and unable to achieve economies of scale, with over 30 firms closing in the past five years. The report recommends focusing on generic drug production as a strategic approach to revitalize the industry. The sustainability of local production relies on enhancing competitiveness, strengthening policy support, and increasing investment incentives. Many of these challenges are shared by other developing and transitional economies [26]. The qualitative findings also noted that pharmaceuticals demand is increasing from year to year. But on the contrary, the local industry output is decreasing, which shows that the local manufacturing companies were not productive. In addition, they marked that the demand increased exponentially, but the supply increased linearly. Around 20% of the country’s needs are covered by local pharmaceutical manufacturers. Similar studies in Ethiopia revealed that less than a quarter of medicines on the National Essential Medicines List are manufactured locally, underscoring the sector’s ongoing heavy dependence on imports [11]. The possible reason may be a lack of hard currency to purchase pharmaceutical inputs (raw materials, reagents, packing materials), and a scarcity of pharmaceutical engineers for calibration and qualification equipment, and utility systems (HAVAC& compressor). In addition, shortage of technology transfers when patents are expired, new generic medicines, poor GMP compliance, turnover of critical staff, and the COVID-19 pandemic. Moreover, the GTP I &II motivation package, as per the policy, is not properly implemented. The product portfolio, which is manufactured by the local pharmaceutical industry, contains almost exclusively old molecules that do not meet the non-communicable disease therapeutics. These were the major challenges for the reduction of local industry output.

In this study, most of the respondents mentioned that the manufacturing premises are of good design and regularly monitored. This finding is almost in line with the WHO’s technical reports [24]. In pharmaceutical manufacturing, quality standards are very stringent as GMP focuses mainly on the manufacturing of safe and quality products [37]. There is a need for improvement as far as the adoption of GMP is concerned for local companies to have their products recognized on the international market. This finding was supported by a study done in Zimbabwe [31]. Regarding quality control practices, local manufacturers carried out written procedures and recorded where necessary, which fulfilled GMP standards. This finding coincides with the results of the WHO critical analysis and the EFDA and PICS guidelines [38]. The finding was also supported by a study conducted in 21 countries [39] and Bangladesh [40]. Concerning sanitation and hygiene, they conduct routine inspections of the premises and utilities and follow GMP standards for cleaning and disinfection practices. This finding coincides with the results of different studies [24,37].

Regarding skill training, in the production processes, the manufacturer firms do have training plans for new personnel, and refresher courses for all personnel to ensure that products of high quality can be produced. This finding was in line with WHO technical reports [24]. It is also supported by the evidence and a study done in India that allow effective training module will give scope to learn and understand the importance of training for personnel in each department to control the human errors caused by lack of proper training system, in general control of all GMP processes in a pharmaceutical manufacturing facility [41,42].

Regarding documentation, local manufacturers have formats and instructions to record the performed activities as per written procedure, and raw material records will be stored for the expected period accordingly. Proper documentation is an important aspect of the quality assurance system and thus should exist for all the processes of GMP. The finding is in line with WHO’s technical reports [24]. Additionally, documentation ensures the accessibility of manufacturing data that is needed for process validation, review, and product statistical analysis [38]. The finding is supported by a study done in India [43].

For local businesses to have their products recognized on the global market, there needs to be an improvement in the implementation of GMP. The major challenges faced by manufacturers’ production were a shortage of foreign currency, technology transfer, a shortage of qualified experts, a lack of locally available raw materials, and the tax system. In addition, operational challenges include power fluctuation as well as internet interruption. These challenges were found mostly to be based on poor implementation of policies and financial constraints. This finding was supported by a study done on the impact of production techniques on the competitiveness of pharmaceutical manufacturing firms in Zimbabwe [31].

Overall to complete the implementation of Africa pharmaceutical manufacturing action plan (PMPA) and establish a robust pharma manufacturing sector in Africa, the continent must redouble efforts in capacity-building, knowledge transfer, cross-sector coordination, and rigorous implementation of the African free trade act, i.e., AfCFTA; further mobilize financial resources from international financial institutions and development banks; and encourage cross-country collaboration to strengthen human capital funding. Only then the continent can close the implementation gap and accelerate the development of Africa’s pharmaceutical industry. Hence, country case studies complement such overall goals in the development of local pharmaceutical manufacturing in Africa.

## 5. Limitations of the study

The study may be subjected to bias from respondents since self-administered questionnaires were used to collect data. Due to the cross-sectional nature of the study, only association was examined, and it was difficult to conclude causation. Our study had a small sample size, which may increase the risk of over fitting and lead to less stable or generalizable estimates. Therefore, future studies with larger sample sizes are recommended to address this limitation.

## 6. Conclusion

Material handling and production planning and machine maintenance were internal factors that have a significant effect on the manufacturing performance of local pharmaceutical firms. Policy (government) and economic factors have a significant effect on the manufacturing performance of local pharmaceutical firms too. The proportion of local pharmaceutical supply was below the expected value, per the government plan, which showed the growth of the pharmaceutical industry being retarded. The policies formulated to support the sector were not yet implemented. The major challenges faced by manufacturers were a shortage of foreign currency, poor technology transfer, lack of qualified experts, lack of locally available raw materials, and the tax system. Hence, the government should support the local pharmaceutical sector by improving access to resources, promoting technology and skills development, supporting local raw material production, and ensuring effective policy implementation. In general, these findings provide actionable evidence for policymakers and industry stakeholders to improve regulatory frameworks, enhance production efficiency, and strengthen the competitiveness of domestic pharmaceutical manufacturers in Ethiopia.

## Supporting information

S1 Text Quantitative survey questionnaire. (DOCX)

S2 Text Qualitative survey questionnaire. (DOCX)
