# Supplementary material for: Assessment of some factors that influence the productiveness and competitive performance of local pharmaceutical manufacturing companies in a low-income country setting: The case of Ethiopia
Source: PLOS Glob Public Health. 2025 Dec 11;5(12):e0005631. doi: 10.1371/journal.pgph.0005631 (PMC12698008; doi:10.1371/journal.pgph.0005631)
Supplement: S1 Text — (DOCX) [file pgph.0005631.s001.docx]

Supplementary material 1: Quantitative study tool

Addis Ababa University

College of Health Sciences,

School Of Pharmacy,

Department of Pharmaceutics & Social Pharmacy Regulatory Affairs MSc Programme

Questionnaire for Industry manager, Production head, QA or R&D managers, Sales and Marketing Senior staffs of Ethiopian local pharmaceutical industries

Dear respondent

My name is YEGNANEH ANLEY; I am a postgraduate student at Addis Ababa University, School of Pharmacy, and currently doing a thesis research work. My study is on the topic “Assessment of the Influence of production techniques and medicines regulation on the competitive performance of local pharmaceutical industries in Ethiopia”. The research is aimed to full fill the partial requirements for the Master of Science degree in medicine regulatory affairs. So this questionnaire is designed to collect the necessary information /data to answer the research question of the thesis. Therefore, I will be grateful if you kindly take time to complete this questionnaire. Participation is voluntarily and you had a right to refuse, interrupt and with draw from participation in this study. However, participation in the research helps to understand the agenda diligently and hence thereof designing possible solutions to the challenges that will help the sector. Please don't write your name on the questioner. All the information in the questioner will be treated in confidentially.

If you had further inquiry you can contact me Yegnaneh Anley (TEL: XXXXX),

Email: yegnaneh2006@gmail.com. My Supervisor Ayenew Ashenef (TEL: XXXX); the School of Pharmacy ERB( 011560212)

Annexes (2): Quantitative Questionnaire

1. Section A: Background Information

Company Profile

A. Company name.........................................................

B. Company Size

No of Employees: A. <100 B. 100-250 C. 250-500

C. Number of current product lines? --------------------

List them ……………………………………………………….………………………

D. What type of production does the company involve in? (Tick all that are applicable).

I. Primary (API or Excipient

II. Secondary (finished product)

--human medicine ,

- Or veterinary medicine

- Both Human and veterinary medicine

III. Tertiary (primary& secondary packaging)

IV. Human Vaccines or veterinary vaccine

V. Drug delivery: capsules

1. Section B: Respondents Profile

1. Respondent sex: Male Female

2. In which of the following age brackets do you belong? Below 21-30 years 31-40 years 41-50 years over 50 years

3. What is your education level (state the highest level?)

A. Diplomas B. Degree, C Master and above D. Other

E. Add field of study:_____________

4. How many years have you been with your employer?

A. Less than 3years B.3- 6years C. 7- 10 years D. over11 years

5. Position

A. Industry Manager B. Production Head C. QA or R&D mangers D. Sales or/and Marketing Senior staff

2. Kindly indicate whether you agree or disagree with the following statements regarding the direct or indirect contribution of your company's performance. (Key: 1:-Strongly disagree, 2:-disagree, 3:-neutral, 4:-Agree, 5:- strongly agree)

2.1. How do you rate the contribution of the following factors on manufacturing downtime which influences the performance of your company?

| S.No | Statements | Rating scale | | | | |
| --- | --- | --- | --- | --- | --- | --- |
|  |  | 1 | 2 | 3 | 4 | 5 |
| 2.1.1 | Lack of reliable electricity service |  |  |  |  |  |
| 2.1.2 | Power fluctuation |  |  |  |  |  |
| 2.1.3 | Raw Material and accessory delay/ shortages |  |  |  |  |  |
| 2.1.4 | Lack of experts on machine maintenance |  |  |  |  |  |
| 2.1.5 | Shortage of timely trend personnel |  |  |  |  |  |
| 2.1.6 | Staff turnover |  |  |  |  |  |
| 2.1.7 | Poor plant laid out to ensure the rapid and smooth movement of material |  |  |  |  |  |
| 2.1.8 | Absence of regular based machine maintenance |  |  |  |  |  |
| 2.1.9 | Unplanned machine maintenance |  |  |  |  |  |

2.2. How do you rate the contribution of production planning and machine maintenance on improving the capacity of your company's manufacturing process?

| S.No | Statements | Rating scale | | | | |
| --- | --- | --- | --- | --- | --- | --- |
|  |  | 1 | 2 | 3 | 4 | 5 |
| 2.2.1 | Market planning on providing quantities and products for demand at the highest level of manufacturing efficiency |  |  |  |  |  |
| 2.2.2. | Production strategy determination to minimize setup costs and holding costs |  |  |  |  |  |
| 2.2.3 | Planning the sequences of work elements involved in each operation |  |  |  |  |  |
| 2.2.4 | Taking of Preventive maintenance of the machine to reduce the chances of break down |  |  |  |  |  |
| 2.2.5 | Presence of regular machine maintenance schedule in your firm |  |  |  |  |  |

2.3. How do you rate the contribution of material handling to improving the capacity of your company's manufacturing process?

| S.No | Statements | Rating scale | | | | |
| --- | --- | --- | --- | --- | --- | --- |
|  |  | 1 | 2 | 3 | 4 | 5 |
| 2.3.1 | Proper handling of the material by using personnel protective gloves |  |  |  |  |  |
| 2.3.2 | Set principles on material handling |  |  |  |  |  |
| 2.3.3 | Implementation of Current Good Manufacturing Practices (CGMP) on Material handling |  |  |  |  |  |
| 2.3.4 | Practicing waste reduction and utilization by all individuals |  |  |  |  |  |
| 2.3.5 | Presence of inventory control |  |  |  |  |  |
| 2.3.6 | Mark the locations of inventory, tools & supplies |  |  |  |  |  |

2.4. How do you rate the contribution of the following factors to upgrade the technology of your company?

| S.No | Statements | Rating scale | | | | |
| --- | --- | --- | --- | --- | --- | --- |
|  |  | 1 | 2 | 3 | 4 | 5 |
| 2.4.1 | In-house Research and Development |  |  |  |  |  |
| 2.4.2 | Obtaining updated technology by donation |  |  |  |  |  |
| 2.4.3 | Increase the habit of updated technology utilization |  |  |  |  |  |
| 2.4.4 | Affiliation with universities and public research institutes |  |  |  |  |  |
| 2.4.5 | Using the services of foreign consultants and movement of skilled labor from abroad |  |  |  |  |  |
| 2.4.6 | Technology transfer through technology licensing, capital goods imports |  |  |  |  |  |

2.5. How do you rate the contribution of various policy and economic factors to the performance of your company?

| S.No | Statements | Rating scale | | | | |
| --- | --- | --- | --- | --- | --- | --- |
|  |  | 1 | 2 | 3 | 4 | 5 |
| 2.5.1 | An overblown of the local tax system |  |  |  |  |  |
| 2.5.2 | Lack of GTP motivation package |  |  |  |  |  |
| 2.5.3 | Government Bureaucracy |  |  |  |  |  |
| 2.5.4 | Financial constraint |  |  |  |  |  |
| 2.5.5 | Shortage of foreign currency |  |  |  |  |  |
| 2.5.6 | Foreign procurement should not weaken local drug manufacturing |  |  |  |  |  |
| 2.5.7 | Inflation rate has influence |  |  |  |  |  |
| 2.5.8 | The general economic climate have influence on the local industry |  |  |  |  |  |
| 2.5.9 | Interest rates have influence on the local industry |  |  |  |  |  |

2.6. How do you rate the contribution of regulatory and legal provisions on the performance of your company?

| S.No | Statements | Rating scale | | | | |
| --- | --- | --- | --- | --- | --- | --- |
|  |  | 1 | 2 | 3 | 4 | 5 |
| 2.6.1 | The presence of pharmaceutical regulatory requirements |  |  |  |  |  |
| 2.6.2 | Increasing establishment and enforcement of GMPs as common standards for quality |  |  |  |  |  |
| 2.6.3 | Corruption and Long processes of registration |  |  |  |  |  |
| 2.6.4 | Lack of Self-inspection or auditing on your company |  |  |  |  |  |
| 2.6.5 | Lack of government inspectorate to ensure adherence by manufacturers to all licensing provisions and GMP |  |  |  |  |  |
| 2.6.6. | The complication of common technical document (CTD) facilitate product registration |  |  |  |  |  |

2.7. How do you rate the following challenges on technology building at your company?

| S.No | Statements | Rating scale | | | | |
| --- | --- | --- | --- | --- | --- | --- |
|  |  | 1 | 2 | 3 | 4 | 5 |
| 2.7.1 | Lack of access to appropriate technology |  |  |  |  |  |
| 2.7.2 | Lack of government incentives for innovation |  |  |  |  |  |
| 2.7.3 | Absence of rules and agencies to promote technology transfer |  |  |  |  |  |
| 2.7.4 | Lack of short-term and long-term training for personnel on technology transfer |  |  |  |  |  |
| 2.7.5 | Lack of access to new Technologies |  |  |  |  |  |
| 2.7.6 | Poor use of new technology/ equipment in your company |  |  |  |  |  |

2.8.If the challenges still persistent what kind of interventions do you think are important to alleviate the challenges? (Tick to those who will apply)

| A | Further investment by your firm |  | |
| --- | --- | --- | --- |
| B | Government support |  | |
| C | Industry-university linkage |  | |
| D  E | Local/foreign collaboration  Others, please specify: |  | |
|  |  | |  |

2.9. How do you rate the threat of new entrants on the performance of your company?

| S.No | Statements | Rating scale | | | | |
| --- | --- | --- | --- | --- | --- | --- |
|  |  | 1 | 2 | 3 | 4 | 5 |
| 2.9.1 | Lack of conducive environment for a pharmaceutical company |  |  |  |  |  |
| 2.9.2 | Lack of access to land |  |  |  |  |  |
| 2.9.3 | Lack of expert on manufacturing and QC area |  |  |  |  |  |
| 2.9.4 | Specialized knowledge in a pharmaceutical manufacturing area |  |  |  |  |  |
| 2.9.5 | Large capital requirement |  |  |  |  |  |
| 2.9.6 | Presence of brand loyalty |  |  |  |  |  |

2.10. How do you rate the contribution of the bargaining power of buyers and suppliers to the performance of your company?

| S.No | Statements | Rating scale | | | | |
| --- | --- | --- | --- | --- | --- | --- |
|  |  | 1 | 2 | 3 | 4 | 5 |
| 2.10.1 | Buyers abilities to force down prices |  |  |  |  |  |
| 2.10.2 | Buyers abilities to switch to competing brands |  |  |  |  |  |
| 2.10.3 | Buyers buying products directly from suppliers influence this industry |  |  |  |  |  |
| 2.10.4 | A large number of suppliers have an impact on this industry |  |  |  |  |  |
| 2.10.5 | The threat of suppliers establishing retail outlets influence this business |  |  |  |  |  |
| 2.10.6 | The strength of suppliers influence this industry |  |  |  |  |  |

2.11. How do you rate the contribution of threats of substitutes and competitive rivalry on the performance of your company?

| S.No | Statements | Rating scale | | | | |
| --- | --- | --- | --- | --- | --- | --- |
|  |  | 1 | 2 | 3 | 4 | 5 |
| 2.11.1 | The presence of new products or equivalent from a competitor |  |  |  |  |  |
| 2.11.2 | The replacements of existing product by new product |  |  |  |  |  |
| 2.11.3 | Consumers abilities to search for new product have influence on this industry |  |  |  |  |  |
| 2.11.4 | High exist barriers of pharmaceutical firms leads to competition in this industry |  |  |  |  |  |
| 2.11.5 | High industry growth rate leads to competitive rivalry in the industry |  |  |  |  |  |
| 2.11.6 | product differentiation among pharmaceutical product leads to competition in this industry |  |  |  |  |  |

2.12. How do you rate the contribution of the following factors whether if apply cost leadership competitive strategy on the performance of your company?

| S.No | Statements | Rating scale | | | | |
| --- | --- | --- | --- | --- | --- | --- |
|  |  | 1 | 2 | 3 | 4 | 5 |
| 2.12.1 | Reducing the prices of goods to attract customers |  |  |  |  |  |
| 2.12.2 | Offering free samples at times |  |  |  |  |  |
| 2.12.3 | Ensuring good customer service |  |  |  |  |  |
| 2.12.4 | Outsourced or discontinued any services which they do not have a low-cost strategy |  |  |  |  |  |
| 2.12.5 | Uses the latest technology to minimize its costs |  |  |  |  |  |

- 1. How do you rate the contribution of the following factors whether if apply differentiation competitive strategy on the performance of your company?

| S.No | Statements | Rating scale | | | | |
| --- | --- | --- | --- | --- | --- | --- |
|  |  | 1 | 2 | 3 | 4 | 5 |
| 2.13.1 | Maintain a strong brand/image identification |  |  |  |  |  |
| 2.13.2 | Invest in creativity & innovation to develop new products |  |  |  |  |  |
| 2.13.3 | Undertakes an advertisement about your company product |  |  |  |  |  |
| 2.13.4 | The company offers a broad product range to cater for varied needs |  |  |  |  |  |
| 2.13.5 | There is innovation in technology to differentiate products |  |  |  |  |  |

2.14. How do you rate the contribution of the following factors whether if apply focus competitive strategy on the performance of your company?

| S.No | Statements | Rating scale | | | | |
| --- | --- | --- | --- | --- | --- | --- |
|  |  | 1 | 2 | 3 | 4 | 5 |
| 2.14.1 | Serves for a specific therapeutic group |  |  |  |  |  |
| 2.14.2 | Serves for a specific customer (e.g. pediatric) segment |  |  |  |  |  |
| 2.14.3 | The firm has special product/service for specific target market |  |  |  |  |  |
| 2.14.4 | Products are offered in lower prices as a focus strategy |  |  |  |  |  |
| 2.14.5 | The firm offers a narrow-limited range of products |  |  |  |  |  |

3. Which of the following factors increase your company's sales? (To select/tick all that applies)

1. The increasing of product portfolio of your company
2. Develop customer-focus sales structure
3. Make work more efficient with technologies
4. Increase sales force knowledge by organizing training and meeting
5. Others, please specify:--------------------------------

4. What were the main internal and external factors which increase the market share of your company? (To select/tick all that applies)

| A | The firm brings to market a new technology its competitors have yet to offer |  |
| --- | --- | --- |
| B | The company strengthening customer relationships and protect their existed market share |  |
| C | The company obtained a GMP certificate |  |
| D | The company built appropriate quality products, affordable price, promotion, and distribution strategies |  |

E. Others, please specify:_______________________________________

5. Does your company invest in innovation and creativity to adapt differentiation strategy?

A. Yes B. No

6. If yes for Q5.Which of the following has a direct contribution to the increment of innovations of your firm? (To select/tick all that applies)

1. Using GMP as a production tool
2. In-house Research and Development
3. Working with universities and public research institutes
4. Appropriate work of Staff trained to apply the technologies
5. Schedule based investment of company for innovation and creativity
6. Others, please specify:---------------------------------------------------------------

7. Is your company profitable?

1. Yes B. No

8. If yes for Q7: Which of the following increase the profitability of your company? (To select/tick all that applies)

1. Because of applied GMP as a production technique tool
2. Proper market competition for domestic pharmaceutical Companies
3. Following the situation of external and internal environment
4. By revising production steps regularly to develop product portfolio
5. Others, please specify:
