# Supplementary material for: Assessment of some factors that influence the productiveness and competitive performance of local pharmaceutical manufacturing companies in a low-income country setting: The case of Ethiopia
Source: PLOS Glob Public Health. 2025 Dec 11;5(12):e0005631. doi: 10.1371/journal.pgph.0005631 (PMC12698008; doi:10.1371/journal.pgph.0005631)
Supplement: S2 Text — (DOCX) [file pgph.0005631.s002.docx]

Supplementary material2: Qualitative survey questionnaire

Supplementary material 1: Quantitative survey questionnaire

**Guide : Interview Questionnaire to Ethiopian local pharmaceutical industry**

I. Part one: personnel information:

Name of your company: _____________________________

Your current position in the company _____________________

Sex: male: __________________Female: ______________________

Age:_____________________________

How long have you been working in the company________________________

Qualification________________________________________________________

1. Section A: Assessment of production techniques using Good Manufacturing Practices (GMP) standards:

1. How do you describe the level of status for essential production techniques components of the good manufacturing practice (GMP) applied in your Organization such as

About manufacturing premises of good design and regularly monitored

………………………………………………………………………………………………………

How do you describe the level of status about the major plant services, systems, and utilities practiced in the manufacture of pharmaceutical products in your organization such as:

Heating, ventilation, and air-conditioning (HVAC) and Lighting suitability

……………………………………………………………………………………………

Water for pharmaceutical use (WPU) utilities,

…………………………………………………………………………………………………

Gases/Compressed air utilities

………………………………………………………………………………………………

About Equipment of good design & properly maintained

…………………………………………………………………………………………………

About the level of quality control (QC) practices status at your organization:

In regards of quality control of raw materials, packaging and finished product

…………………………………………………………………………………………………

How do you describe the application level of sanitation and hygiene practiced in the manufacture of medicine products in your Organization such as:

About correct choice of cleaning and disinfection to monitor regularly the premises and utilities

………………………………………………………………………………………………………

About personals sanitation and hygiene practiced by your plant

………………………………………………………………………………………………………

How do you describe the level of training to qualified Personnel on various skills and hygiene training practiced in the manufacture of pharmaceutical products at your organization?

………………………………………………………………………………………………………

How do you describe the level of practice on the written procedure and documentation adopted in the manufacture of medicine products at your organization?

……………………………………………………………………………………………………… What challenges are you facing in adopting these production techniques when you applied good manufacturing practice (GMP)?

…………………………………………………………………………………………………

Has the company ever gone through a product recall in the last five years, if so, what was the cause of the recall(s)?

……………………………………………………………………………………………..........

Has the company procedure for complaint/ recall handling systems?

……………………………………………………………………………………………………… Does your organization get GMP compliance certificate from Ethiopian feed and drug regulatory authority (EFDA) ……………………………………………………………………

II. Section B: Assessment of strategic plan and policy implemented by local pharmaceutical industry

1. Growth and Transformation Plan One (GTP I):

As local pharmaceutical manufacturer do you know the GTPI motivation package? List the packages with expected outcome benefits?

............................................................................................................................................................

As the local manufacturer do you utilize the motivation input provided by the government during the growth and transformation plan one (GTP I &II) to increase the manufacturer market share? If so how to evaluate the outcome

….…………………………………….…………………………………………………………

Is there any constraint to utilize motivation input to improve productivity of the local pharmaceutical industry proved by the government according to GTPI &II policy objective? If so describe these challenges?

......................................................................................................................................................

2. As the pharmaceutical manufacturing company did you follow marketing strategy to increase your market share? Yes NO

3. If yes for Q2 which type of competitive advantage strategy did you follow?

4. As local pharmaceutical product manufacturer are you participate on the donor procurement products such as for communicable diseases (Malaria, HIV/AIDS and TB, etc.) therapeutic product? Yes No

5. If your answer is No for Q6 describe the reason why not participated for the donor procurement?

……………………………………………………………………………………………

III. Section C: The Main Operational Challenges in Pharmaceutical production

1. 1. As local pharmaceutical manufacturer how much your manufacturer production productivity influences by the fowling bottlenecks?

How human resource constraints include trained and licensed pharmacists; people with expertise in chemistry, pharmacology, and physical, chemical and biological sciences; technicians and engineer challenges the production of your plant?

........................................................................................................................................................… How poor infrastructure including unreliable basic services such as water and electricity does challenges the production of your company?

………………………………………………………………………………………………………How lack of collaborative linkages: between various relevant ministries, departments, and institutions, challenge the production of your company?

………………………………………………………………………………………………………

How high cost of finance from the perspective of interest rates, hard currency and exchange rate fluctuation and the loan periods challenge at your company?

………………………………………………………………………………………………

Annex 4: GUIDE II: Interview Questionnaire to Ethiopian Food and Drug Authority (EFDA)

I. Part one: personnel information:

Name of your company: _____________________________

Your current position in the authority _____________________

Sex: male: __________________Female: ______________________

How long have you been working in the authority __________________ Qualification_---------

1... Does the domestic companies compliant to applied essential production techniques component in good manufacturing practice (GMP)?

…………………………………………………………………………………………………

2. Does the authority / recognize the advantage of strengthening the enforcement of regulation on the sub-standard product to develop a quality product portfolio in local pharmaceutical industries? Describe the activities

………………………………………………………………………………………………

3. What is the minimum requirement to get market authorization for new generic medicine product registration application requested by local pharmaceutical manufacturer?

………………………………………………………………………………………………

4. As regulatory authority what is the regulatory requirement to waive biopharmaceutical studies reports for generic local products registration manufactured by local pharmaceutical manufacturer?

…………………………………………………………………………………………………

5. As regulatory authority what is the minimum requirement requested to provide stability study data for registration application to get evidence for the determination of the shelf life of FPP with time …………………………………………………………………………………………..

6. Does authority notices any company that has ever gone through a product recall in the last five years, from the quality defect perspective and as result of quality defect revokes the market authorization?

………………………………………………………………………………………………

7. Is there procedure to approved quality of the Product at batched base approach or only during registration only?

…………………………………………………………………………………………………

8. As regulatory body did you facilitate the appropriate materials such as the reference slandered and reagents for the local pharmaceutical product quality test of finished product ………………

…………………………………………………………………………………

**ANNEX 5: GUIDE III: Questionnaire to Food, Beverage, and Pharmaceuticals industry development institute (FBPIDI)**

I. Section A: assessment of the various policies on the privilege of local pharmaceutical industry goals implementation:

1. The objective of your institute is to accelerate technology transfer, achieve transformation and enable the industry to be competitive at the international level. So Describe your participation how the local pharmaceutical manufacturer implements to achieves the drug, health, and industry policy?

............................................................................................................................................

2. Does your institution formulate policies, strategies, and action plans that assist in the acceleration of the domestic product to cover the country's medicine demand by the local pharmaceutical manufacturer based on the drug & health policy objective?

............................................................................................................................................................

3. Growth and Transformation Plan One (GTP I):

Does your institution know the GTPI motivation package; List the packages with expected outcome benefits?

............................................................................................................................................................

As an industrial supporter are you working with the local manufacturer to facilitate the utilizing of the motivation input provided by the government during the growth and transformation plan one (GTP I &II) to increase market share? If so how to evaluate the outcome

….…………………………………….……………………………………………………………

As an industrial supporter is there any constraint to utilize motivation input to support the local pharmaceutical industry proved by the government according to GTPI &II? If so how did you fix it?...................................................................................................................................

GTP I and II are already recently passed, but from this strategic plan as an industrial supporter did you learn the sector failures and non-achievements? What were those?

…………………………………………………………………………………………………

As an industrial supporter is there an implementation on pharmaceutical manufacturing development strategic gold planning or not in the present and coming years?

…………………………………………………………………………………………

4. Investment and industrial development environment, how strong is the country's financial sector in supporting the pharmaceutical industry and what are the requirements to access such financial support?

......................................................................................................................................................

5. Does the domestic company compliant with good manufacturing techniques component in production? If so what challenges are you notice when the local manufacturers are adopting good manufacturing practices as production techniques?..........................................................................

6. How your institutions work to increase quality product portfolio in the local pharmaceutical industry?

...................................................................................................................................................

7. How the government solves constraints to access foreign expertise by domestic policies that restrict the employment of foreigners? . ...................................................................

If it is not allowed the employment of foreign experts, do you negotiate with the government to reform these regulations so that local firms can use the services of foreign experts over a significant period? What was the measure?

............................................................................................................................................................

8. As an industrial supporter institute do you coordinate domestic companies to work with bi-lateral donors (international cooperation such as UNIDO, UNCTAD, WHO) to support special projects for upgrading production capacity for medical products? Describe any activities initiated by you ……………………………………………………………………………….

9. Pharmaceutical firms do not operate in Poor infrastructure as favorable environments like cluster technology parks or special economic zones. So as industrial supporter institute what is your contributions to the local manufacturer for decrease unfavorable environments which increase their basic operational costs?

...............................................................................................................................................

…………………………………………………………………………………………………

**Annex 6: GUIDE IV: Questionnaire for Ethiopian pharmaceuticals and medical supplies manufacturing association**

1. Section A: Assessment of the various policies on the privilege of local pharmaceutical industry goals implementation from the perspective of the association

1. As the association describes the government support how to implement the drug, health, and industry policy by creating a conducive environment to achieve their mission and goals of local pharmaceutical industries

…………………………………………………………………………………………………

2 .Does the association works strategically to facilitate the country's pharmaceutical product demand covered by the local pharmaceutical manufacturer based on the drug policy objective? Describe.

. .........................................................................................................................................

3. As the association describes to what extent the local companies contribute to the national economy by saving hard currency by replacing import product and creation of job opportunity in the perspective of drug policy integrated industry policy?

...................................................................................................................................................

4. According to the health policy the government increased the health coverage 95% since 2011 GC; as association did you believe due to this the pharmaceutical demand increase?

…………………………………………………………………………………………………..

From this market opportunity how much local pharmaceutical company did use the policy advantage to increasing their market share?

......................................................................................................................................................

If there is any obstacle to utilize this market opportunity, please describe it as an association?

…………………………………………………………………………………………………

5. Growth and Transformation Plan One (GTP I&II):

As an association, the domestic manufacturer utilizes the motivation input provided by the government during the growth and transformation plan (GTP I&II) to increase market share? Described the change by this motivation input

….…………………………………….…………………………………………………………

As association did you identified any constraint to utilize motivation input to support the local pharmaceutical industry proved by the government according to GTPI&II? If so list them?

......................................................................................................................................................

6. From the perspective of industry policy, AS association does the local company get support from the sector minister to achieve the member company goals? , describe the support?

......................................................................................................................................................

7. As an association how you understand the country's financial sector in supporting the pharmaceutical industry and what are the requirements to access such financial support from the perspective of investment and industrial development environment?

................................................................................................................................................

8. As the association what is your support for the domestic company compliant with good manufacturing techniques component in pharmaceutical production? ………………………….

2. Section B: Assessment of the industry forces to competitiveness of local Pharmaceutical industry from the market share and quality product supplied access perspective

1. As an association describes the influence of intensity of rivalry among the existing local pharmaceutical industry, do you recognize quality defects on the product due to the greatness of competition as a result of the price reduction?

……………………………………………………………………………………………..

2. As the association describes the influence of supplier’s large number, their product supplier's strength and establishing its retail outlet's influence on this industry market share and competitiveness?

.......................................................................................................................................................

3. Section C: Assessment of the Bottlenecks in Local Pharmaceutical Production

1 As association how did you work strategically to mitigate the influence of productivity of the local pharmaceutical manufacturer production by the fowling bottlenecks?

1.1. Human resource constraints include trained and licensed pharmacists; people with expertise in chemistry, pharmacology, and physical, chemical, and biological sciences; technicians and engineer?.................................................................................................

1.2. As association list unfavorable environments or poor infrastructure which increase their basic operational costs?

…………………………………………………………………………………………………

1.3. As association described lack of collaborative linkages such as between various relevant ministries, departments, and institutions which influence the productivity of local pharmaceutical manufacturer?

…………………………………………………………………………………………………

1.4. As association described the high cost of finance from the perspective of, interest rates, cost of a manufacturing plant, and the loan periods?
